# Delayed buccal fat pad herniation: An unusual complication of buccal flap in cleft surgery

**DOI:** 10.4103/0970-0358.53019

**Published:** 2009

**Authors:** Puneet Tuli, Atul Parashar, Vipul Nanda, Ramesh K. Sharma

**Affiliations:** Department of Plastic Surgery, Postgraduate Institute of Medical Education and Research, Chandigarh, India

**Keywords:** Buccal musculomucosal flap, Cleft palate, Herniation of buccal fat pad

## Abstract

Buccal musculomucosal flap is commonly used in cleft palate surgery for providing additional lining when nasal mucosa is inadequate. We report an unusual complication of progressively increasing fat herniation from the sutured donor site which started appearing on the third postoperative day. This necessitated excision of the protruding fat pad on the seventh postoperative day. The possible mechanism and precautions for prevention of this complication are discussed.

## INTRODUCTION

Buccal flap for cleft repair was first described by Mukherji *et al.*, in the year 1969.[1] Since then, many variations and different uses of this flap have been reported by several authors. [2–9] Many complications like swelling of the face, infection, stenosis of the parotid duct and difficulty in the eruption of molar teeth have already been described in the literature.[3,4,6,8] We hereby report an unusual complication in which a delayed herniation of the buccal fat occurred at the flap donor site.

## CASE REPORT

**Figure 1:**
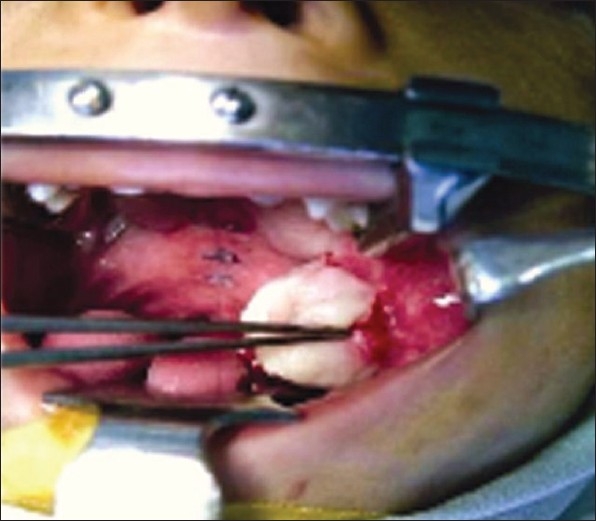
Herniating buccal fat; forceps' tip at the exit point

A six-year-old male child, follow-up case of unilateral complete Group 3 cleft was admitted for repair of the cleft palate. On examination cleft was 13-mm wide and intertuberosity distance was 23 mm and a buccal flap was planned for reconstruction of the nasal layer. Flap was based posterior to the maxillary tuberosity and was 1.5 cm wide and 6 cm long. The flap was raised as a musculomucosal flap and closure of the donor site was done using chromic catgut. Postoperatively, the oral examination was unremarkable. However, a small protrusion of the fat at the donor site was noticed on the third post operative day which then progressively increased over the next four days. The patient had significant herniation on the seventh day [Figure 1] for which he was taken to the operating room. Excision of the fat and closure of the defect was done with 3-0 Polyglactin 910 (Vicryl^®^) under general anaesthesia. At one month follow-up the suture lines were fully healed.

## DISCUSSION

Buccal flaps have been used in palatal surgery for lengthening of the nasal layer, reconstruction of the poor nasal layer repair and to prevent reattachment of the levator sling on the hard palate.[6] This flap can be raised either as a mucosal or a myomucosal flap and is usually based near the anterior pillar of the fauces.[1] The unusual complication of delayed buccal fat herniation which we faced needs to be understood and avoided. Anatomically, buccal fat is described as consisting of a central body and four extensions - pterygoid, buccal, superficial and deep temporal.[10] The main body is situated deeply along the posterior maxilla and upper fibres of the buccinator muscle. It is this portion of the fat which herniates through donor site when a buccal flap is raised. Functionally, the buccal fat represents a specialized type of fat also known as *syssarcosis*, fat that enhances intermuscular motion.[10] In our case, though no herniation of the fat was noted in the immediate and early postoperative period, some extrusion must have occurred on the third postoperative day due to the syssarcosis function of the buccal fat. This minimal extrusion might have progressively increased due to the relentless pushing by muscles of mastication; the rent in the suture line acting as a ball-valve preventing the herniated fat from reducing back. We suggest that while closing the donor site, special care should be taken to prevent the herniation of the fat pad and sutures should be placed in close proximity to avoid delayed extrusion as well.

## CONCLUSION

Minimum donor site morbidity and complication makes the buccal flap a useful armamentarium of a cleft surgeon. However, meticulous closure of donor site is important to prevent delayed extrusion.
